# Comparative measurement of short-term fluoride release and inhibition of caries around restoration by ion releasing restorative materials: an in vitro study

**DOI:** 10.1038/s41598-024-78918-x

**Published:** 2025-01-10

**Authors:** Eman T. El-Adl, Maha M. Ebaya, El-Sayed E. Habib, Nadia M. Zaghloul

**Affiliations:** 1 Conservative Dentistry Department, Faculty of Dentistry, Mansoura University, Mansoura, Egypt; 2 Microbiology and Immunology Department, Faculty of Pharmacy, Mansoura University, Mansoura, Egypt

**Keywords:** Fluoride release, Ion-releasing materials, Cariogenic bacteria, Secondary caries

## Abstract

The main objective of the current study is to compare short-term fluoride release of three ion releasing restorative materials and assess their inhibitory effect on secondary caries. Materials used in this study included, Self-adhesive hybrid composite (group A), Ion releasing flowable composite liner (group B), and alkasite restorative material (group C). Twenty-two discs were fabricated from each material for short-term fluoride release test, conducted on days 1, 7, and 14. For assessing secondary caries inhibition, sixty-six sound molar teeth were used and standardized class V cavities were prepared. Teeth were divided into three groups according to each material, followed by 800 cycles of thermocycling. Subsequently, teeth were immersed in a solution containing cariogenic bacteria for 30 days. After that, teeth were sectioned bucco-lingually and analyzed using a polarized light microscope to measure inhibition area, outer lesion depth, and extension. Data was statistically analyzed using different tests. The study results revealed a statistically significant differences in fluoride release existed among materials. Self-adhesive hybrid composite exhibited the highest fluoride release. Lesion extension and depth were statistically significantly greater next to Ion-releasing flowable composite liner. The inhibition areas next to the Self-adhesive hybrid composite were statistically significantly larger than the other two materials. In conclusion, all tested ion-releasing restorative materials displayed fluoride release and the potential to inhibit secondary caries formation. Self-adhesive hybrid composite demonstrated the highest fluoride-releasing potential and the greatest ability to inhibit secondary caries. Conversely, Ion-releasing flowable composite liner exhibited the least fluoride release with minimal secondary caries inhibition. Increasing fluoride release correlated with larger inhibition areas and reduced outer lesion depth and extension.

## Introduction

Significant advancements in resin composite formulations have been made over the past decade to address clinical challenges. Bulk-placement techniques, innovative filler formulations, and streamlined adhesion protocols have resulted in more user-friendly applications^1^. Despite these advancements, clinical issues such as technique sensitivity, polymerization shrinkage, and the absence of antibacterial properties persist. As a result, secondary caries and bulk fractures continue to be the primary causes of failure^2^.

Secondary caries, also referred to as caries associated with restorations or sealants (CARS), is defined as carious lesions occurring at the margins of existing restorations^3^. The complexity of caries around restorations arises from its multifactorial nature, which involves both the pathological process of primary carious lesions and the impact of various restorative materials and their formulations^4^. Studies have shown that biofilm tends to accumulate more thickly around resin composite restorations compared to glass ionomer restorations^5^. In vivo plaque studies have revealed that lactic acid-producing bacteria are found in significantly higher concentrations around resin composite restorations than around amalgam or glass ionomer restorations^6^. Consequently, fluoride-releasing materials with remineralizing and antibacterial properties have become more popular in recent years as they offer the potential to reduce the risk of secondary caries development^7^.

Conventional glass ionomer cements (GICs) and their advancements, such as high-viscosity glass ionomer (HV-GIC), resin-modified glass ionomer (RMGIC), and compomers, are among the most commonly used fluoride-releasing restorative materials. Fluoride ions can saturate the liquid environment around the restorations, promoting the enhanced precipitation of CaF2 crystals^8^. However, a key limitation of GIC is its low fracture toughness, which restricts its use to low load-bearing areas like the buccal and lingual surfaces. Modifications, including an increased powder-liquid ratio and changes in chemical composition, have demonstrated improvements in physical properties and higher clinical longevity^9^. A potential solution to address the limitations of GIC is to incorporate resin composite restorations, which possess superior mechanical properties compared to GIC, with reactive fillers that protect the tooth against secondary caries^8^. Additionally, with the rise of minimally invasive dentistry, there is an increasing demand for therapeutic ion-releasing materials capable of interacting with underlying tissues and enhancing the longevity of the material-dentine interface^7^. The release of therapeutic ions from dental materials can help suppress residual infections, particularly in partial and selective caries removal techniques, and improve the survival rate of restored teeth^10^.

To date, several new commercially available ion-releasing composites with claimed bioactivity have been introduced, like Regen Flowable Composite liner (Vista Apex, USA), Cention Forte (Ivoclar Vivadent, Schaan, Liechtenstein), and Surefil One (Dentsply-Sirona, Charlotte, North Carolina, USA). It is worth noting that recently developed composite materials, such as Cention forte are gaining popularity due to their cost-effectiveness. Cention Forte, classified as an "alkasite," contains an alkaline glass filler capable of releasing acid-neutralizing ions. This material is primarily self-curing but also offers an optional light-curing feature. As a dual-cured restorative material, it can be used as a full-volume replacement in restorations^11^. These recent materials lack enough studies regarding fluoride release potential and their ability to inhibit secondary caries formation. Therefore, there is an increasing need for further studies.

## Materials and methods

This study employed three ion releasing materials, Self-adhesive hybrid composite, Ion releasing flowable composite liner and alkastie restorative material (Table 1).

**Table 1:** Materials used in the study.

Material	Manufacturer	Type	Composition	Batch Number
Surefil One	Dentsply, Konstanz, Germany	Dual-curing, Self-adhesive composite hybrid	MOPOS, BADEP, acrylic acid, stabilizer, .water, reactive glass filler, non-reactive FAS glass filler and initiator	2104000853
Re-Gen Flowable Composite	Vista Apex, USA	Light-curing, Flowable composite liner	Barium-based bioglass, 2-Propenoic acid, 2-methyl-, (1-methylethylidene) bis[4,1-phenyleneoxy(2-hydroxy-3,1-propanediyl)]ester, TEGDMA, Submicron Silica, Benzoic acid, 4-(dimethylamino)-, ethyl ester	90648
Cention Forte	Ivoclar Vivadent, Liechtenstein	dual-curing Alkasite restorative material	UDMA, initiator, pigments, reactive silanized FAS glass filler	740832WW
Re-Gen Universal Adhesive	Vista Apex, USA	Universal Adhesive	2-Propenoic-acid,2-methyl-,(1-methylethylidene)bis[4,1-phenyleneoxy(2-hydroxy-3,1-propanediyl)]ester,1,2,4,5-BTC,1,4-bis[2-[(2-methyl-1-oxo-2-propen1-yl)oxy]-1-[[(2-methyl-1-oxo-2-propen-1-yl)oxy]methyl]ethyl] ester, 10-MDP , Benzoic acid, 4-(dimethylamine)-, ethyl ester, 2-HEMA, Ethyl alcohol	90637
Clean & boost	Vista Apex, USA	Dentin and enamel cleanser	2-hydroxyethyl methacrylate, Propan-2-ol, Nitric acid	90417
Cention Primer	Ivoclar Vivadent, Liechtenstein	self-etching and self-curing primer	2-HEMA, Bis-GMA, ethanol, MA-PA-Ester, 1,10-decandiol dimethacrylate, MA-MO-*PAA*, camphor quinone, 2-DMAM	740832WW
Tryptic Soy Broth	Difco-Becton, USA	Soybean-casein Digest Medium	Bacto™ Trypton, Bacto Soytone, Glucose, Sodium Chloride, Dipotassium Hydrogen Phosphate	8141955

Abbreviations: MOPOS: Modified polyacids, BADEP: Bifunctional acrylate, TEGDMA: Triethylene glycol dimethacrylate, UDMA: Urethane Di methacrylate, BTC: Benzenetricarboxylic acid, MDP: Methacryloyloxydecyl dihydrogen phosphate, HEMA: Hydroxyethylmethacrylate, Bis-GMA: bisphenol A-glycidyl methacrylate, MA-PA: methacrylated phosphoric acid, MA-MO-PAA: methacrylate modified polyacrylic acid, DMAM: dimethylaminoethyl methacrylate.

### Sample size

Sample size was calculated using G*Power software (v3.1.9.7)^12–15^, Universität, Kiel, Germany with an anticipated large effect size (f = 0.4) for fluoride release and anti-cariogenic effects among three groups. Each group comprised 22 samples in a one-way ANOVA, totaling 66 units. This achieved 82% power to detect mean differences at a 0.0500 significance level, with an effect size (f) of 0.4000^16^.

### Fluoride release test

Sixty-six discs (2mm thickness, 10 mm diameter) were prepared from Surefil One, Re-Gen Flowable Composite, and Cention Forte. Discs were fabricated by molds placed on a mylar strip and a glass slab and then another mylar strip and glass slap were used to exert adequate pressure and remove excess material. Constant weight of 0.5 kg was applied to all discs^17^. A total of 22 discs were made for each material. Surefil One composite capsule was activated and placed in a capsule mixer for 10 s, dispensed into the mold and light cured for 20 s from both sides of the glass slaps. Re-Gen Flowable Composite was injected into the mold and light-cured for 20 s. Cention Forte capsules were activated, mixed for 15 s, injected into the mold, and light cured for 15 s from both sides of the glass slaps. After complete setting of the materials, the discs were removed from molds and polished using impregnated aluminum oxide discs (Sof-Lex, 3M ESPE). Sequence of coarse, medium, fine and superfine grain disks attached to low-speed handpiece (NSK, Tokyo, Japan), was done^18^.

Each disc was placed in a separate glass container containing 5mL of deionized water, stored at 37°C in an incubator (Imperial II Incubator, Lab Line Instruments, Inc.) for 24 h, and the water was changed every 24 h. After 24h, each container was agitated, and water was removed for examination. Discs were replaced in 5mL of new deionized water. Fluoride release was measured at day 1, 7 and 14^19^. The water removed from each container was combined with 0.5 mL of Total Ionic Strength Adjustment Buffer III solution (TISAB III). This was done to regulate the pH, prevent the creation of fluoride complexes, and enable accurate determination of the overall fluoride concentration^20^. Fluoride was measured in water using the SPADNS colorimetric method (SM4500 F standard method for examination of water and wastewater 24th edition 2023).

The analysis was based on the reaction between fluoride and a zirconium-dye lake. To create the SPADNS reagent, 958 mg of SPADNS (Sulfanilic acid azochromotrop, Sigma Aldrich, USA) was dissolved in deionized water and diluting it to 500 ml deionized water. Zirconyl-acid reagent was prepared by dissolving 133 mg zirconyl chloride octahydrate, ZrOCl2·8H2O (Sigma Aldrich, USA), in about 25 mL deionized water, adding 350 mL conc HCl, and diluting to 500 mL with deionized water. Acid zirconyl-SPADNS reagent was prepared by mixing equal volumes of SPADNS solution and zirconyl-acid reagent. Standard solutions with fluoride concentrations (0.0, 0.2, 0.4, 0.6, 0.8, 1.0 ppm) were prepared from a certified reference material of fluoride (Merck KGaA, Darmstadt, Germany) and used for calibration. The absorbance of the standards was determined at a wavelength of 570 nm using a UV–visible double beam spectrophotometer (LAMDA 365, PerkinElmer, USA). Samples were diluted by a ratio of 1:50 and measured against the prepared standards to obtain results^21^.

### Evaluation of inhibition of secondary caries caused by cariogenic bacteria teeth selection

Sixty-six sound human molar teeth were collected from periodontal disease patients. These teeth were devoid of calculus, cavities, or defects and underwent thorough cleaning using an ultrasonic scaler. Afterward, they were stored in 0.5% chloramine-T for 48 h and preserved in regularly changed distilled water. All procedures followed infection control standards and regulations that had been approved by the Faculty of Dentistry, Mansoura University, Egypt, Ethical Committee. Ethical approval number is M16020822. Informed consent was taken from all participants. All methods were performed in accordance with the Declaration of Helsinki guidelines and regulations. The molars were divided into three main groups (n = 22) based on the chosen restorative material^22^.

### Teeth preparation

Class V cavities were prepared on the middle third of buccal and lingual surfaces of each tooth^23^, measuring 4mm-long mesiodistally, 2mm-wide occluso-cervically, and 2mm deep^24^. The occlusal and gingival margins were situated on enamel as adhesion to enamel is better and more predictable. Preparation was done using per-marked, (with permanent marker), diamond tips (SF-31SC, Mani, INC, Japan) in a high-speed handpiece (Sirona, Germany) with copious air–water cooling spray, and burs were replaced every fifth preparation^25^. A metal stamp (with the desired dimentions), with handle was used to standerdize cavity preprations. Cavity depth was verified with a graded periodontal probe for accuracy^26^, and all preparations were conducted by one operator.

### Restorative techniques

Regarding Group A, Surefil One capsule was activated and placed in a capsule mixer for 10 s. The material was dispensed into the cavity and it was gradually withdrawn. A translucent matrix (Hawe, soft transparent cervical matrices, Kerr, 67771940) was applied to aid in contouring, and the material was light cured for 10 s through the matrix and for 20 s after matrix removal.

In Group B, the Re-Gen Flowable Composite application involved several steps. First, Clean & Boost was applied to the tooth to the cavity, agitated for 10 s, rinsed thoroughly, and left moist. Following this, the Re-Gen Universal Adhesive System was applied. This involved applying one drop of the adhesive to the mixing well, followed by using a brush to coat one layer of the adhesive over the cavity preparation. It was left for 20 s without drying, and two more coats were applied using the same brush, with the remaining material in the well. The surface was then dried until a thin immobile layer was achieved. Subsequently, the adhesive was light-activated for 10 s using a curing light (C02-C, Premium Plus, USA). Finally, Re-Gen Flowable Composite Liner was applied into the cavity preparation, aided using a translucent matrix for contouring. The restoration was completed by light-activating for 10 s throught the matrix and for 20 s after matrix removal.

For Group C, the procedure involved conditioning of the cavity using Cention Primer. One drop of self-etching self-adhering and self-curing Cention Primer was dispensed into a dipping dish, and a Cention Primer single-use applicator coated with the catalyst was used to stir the liquid for 5 s. The primer was then applied to the cavity, starting from the enamel and moving towards the rest of the cavity. The primer was agitated for 10 s, dispersed with water and oil-free compressed air until a glossy thin immobile layer remained. Next, the Cention Forte capsule was mixed by a capsule mixer for 15 s, and the material was injected into the cavity using a capsule applicator. Contouring was facilitated using a translucent matrix, and the restoration was light cured for 10 s through the matrix and for 15 s after removal of the matrix.

Following the restoration process in all groups, additional steps included finishing and polishing the restorations using impregnated aluminum oxide discs (Sof-Lex, 3M ESPE), were done^27^. Sequence of coarse, medium, fine and superfine grain disks attached to low-speed handpiece (NSK, Tokyo, Japan). Each disc was applied to the restoration for 10 s under water cooling. The same sequence was done for all restorations^28^. Teeth underwent 800 thermocycles at 5°C and 55°C distilled water baths^24^. And an acid-resistant nail varnish was applied to the restored teeth, leaving a 2mm free zone around the cavity margins. Steel wires were affixed to the root portions of every tooth^29^. The tooth/wire units were sterilized using Ultraviolet Radiation for 30 min in MaXtreamTM V (DAIHAN Scientific, Seongbuk-gu, Seoul, Korea)^30^.

### Bacterial system

A standard strain of Streptococcus Mutans (ATCC 25175)^31^, was used for this in vitro study to induce recurrent caries. The purified isolates were underwent short-term and long-term preservation procedures^32^. After that, bacteria was incubated (37°C for 24 h) in Tryptic Soy Broth (TSB) (Difco-Becton, Dickinson, and Company - Sparks, MD, USA) with 5% sucrose to stimulate bacterial growth. The bacteria were then plated in a solid culture medium (TSA, Difco-Becton, Dickinson, and Company - Sparks, MD, USA) to obtain isolated colonies and then incubated (37°C) for 24 h. Afterwards, the formed colonies were transferred to tubes containing TSB and 5% sucrose, starting a preconditioning of the bacteria with sucrose for 6 days. The tube containing the inoculum broth was obtained with approximately 1–2 × 10^6^ colony-forming units (CFU)/ml using portable spectrophotometer (Biochrom, Colorwave WPA, CO7500) at a wavelength of 600 nm^24^.

### Measuring culture density

Cuvettes were cleaned with deionized water. A control solution (blank) was prepared with 1 mL of TSB (with 5% sucrose) in one cuvette. In another cuvette, 1 mL of inoculum broth was loaded (Sample). The spectrophotometer’s wavelength was set to 600 nm^24^, and readings were recorded for the blank and sample cuvettes. The inoculum broth was diluted with TSB (with 5% sucrose) until the culture density reached 0.1^33^. Sixty-six falcon tubes were prepared, each containing a tooth immersed in 5 mL TSB + 5% sucrose and 200 μL of inoculum broth. These tubes were then placed in an anaerobic jar^34^, and incubated for 48 h. Fresh falcon tubes with the same solution were prepared every 48 h, and teeth were transferred to them. Additionally, random old falcon tubes underwent Gram-Stain contamination tests during the one-month procedure. The Streptococcus Mutans strain stained violet, indicating it is Gram-positive^35^.

The measurement of culture density and contamination tests using Gram stain were repeated every 48 h for 30 days. Following this duration, the teeth were removed from the bacterial system, and the steel wires were taken off. Then, each tooth was embedded in epoxy resin. After that, the teeth were sectioned bucco-lingually passing through both restorations, using an Isomet 4000 linear precision cutting machine (Buehler, Lake Bluff, IL, USA), resulting in sections of 500 μm in thickness.

Two random sections were further processed by hand grinding to approximately 100 μm thickness using sandpaper numbered 150, 220, 400, and 600 (Qingdao Shangli Abrasives Co., Ltd.). After that, sections were embedded in water and examined under a polarized light microscope (Olympus BX50; Olympus, Tokyo, Japan) coupled with a digital camera (Olympus Imaging Crop, Model No. E420 DC 7.4 V) to capture and process images. Measurements were recorded in both the occlusal and cervical regions, encompassing Extension (E) from one outer lesion margin to the opposite margin, Depth (D) at the lesion’s deepest point, and Inhibition Area (IA), which represents the distance between the lesion and the restoration margin^24^.

## Results

Data were entered and analyzed using IBM-SPSS software (IBM Corp. Released 2020. IBM SPSS Statistics for Windows, Version 27.0. Armonk, NY: IBM Corp). Quantitative data were initially tested for normality using Shapiro–Wilk’s test with data being normally distributed if p > 0.050. The Kruskal–Wallis H-test was used to compare non-normally distributed quantitative data between multiple groups. For statistically significant result, Pairwise comparisons with Bonferroni correction for multiple tests were performed. The Friedman’s test was used to compare non-normally distributed quantitative data that are measured over multiple time points. For statistically significant result, Pairwise comparisons with Bonferroni correction for multiple tests were performed. The Spearman’s correlation test was used to determine whether there is a linear relationship / association between two non-normally distributed quantitative data. The strength of association was considered low, medium, or high if the correlation coefficient (r_s_) was (> 0.1–< 0.3), (0.3–< 0.5), or (0.5 or more), respectively.

### Fluoride release test

Kruskal–Wallis H-test revealed a statistically significant difference in fluoride release between the three materials on each of the three days. So, Pairwise comparisons were performed and revealed a statistically significantly higher fluoride release in Self-adhesive hybrid composite > Alkasite restorative material > Bioactive flowable composite liner on each of the three days (Table 2).

**Table 2:** Comparisons of fluoride release between the three restorative materials on each day (ppm).

Day	Material						Pairwise comparisons of fluoride release between material
	Group A		Group B		Group C		
	Mdn	IQR	Mdn	IQR	Mdn	IQR	
1	39	34.6–50	3.46	2.4–5.8	11.7	10–12.6	a = < 0.001 b = < 0.001 c = < 0.001
7	15.9	12.5–23	1.66	1.3–2.6	3.96	3.2–4.3	a = < 0.001 b = 0.001 c = < 0.001
14	5.4	4.9–5.7	0.46	0.29–1.4	1.9	1.4–1.95	a = 0.011 b = < 0.001 c = < 0.001
Pairwise comparison of fluoride release between time point	A = 0.003 B = 0.003 C = 0.003		A = < 0.001 B = < 0.001 C = < 0.001		A = 0.003 B = 0.003 C = 0.003		

Notes: Group A: Self-adhesive hybrid resin composite. Group B: bioactive flowable composite liner. Group C: Alkasite restorative material. Mdn = Median. Sig. = significance (p-value). IQR = interquartile range presented as Q1 and Q3. The test of significance is the Kruskal–Wallis H-test.

A: represent significant difference in Day 1 vs. 7, B: represent significant difference in Day 1 vs. 14, C: represent significant difference in Day 7 vs. 14.

a: represent significant difference in Group A vs Group B, b: represent significant difference in Group A vs Group C, c: represent significant difference in Group B vs Group C.

Friedman test revealed a statistically significant difference in fluoride release between the three time points in each of the three materials. So, Pairwise comparisons were performed and revealed a statistically significantly higher fluoride release at day 1 > day 7 > day 14 in each of the three materials (Table 2).

### Evaluation of inhibition of secondary caries caused by cariogenic bacteria

**Fig. 1:**
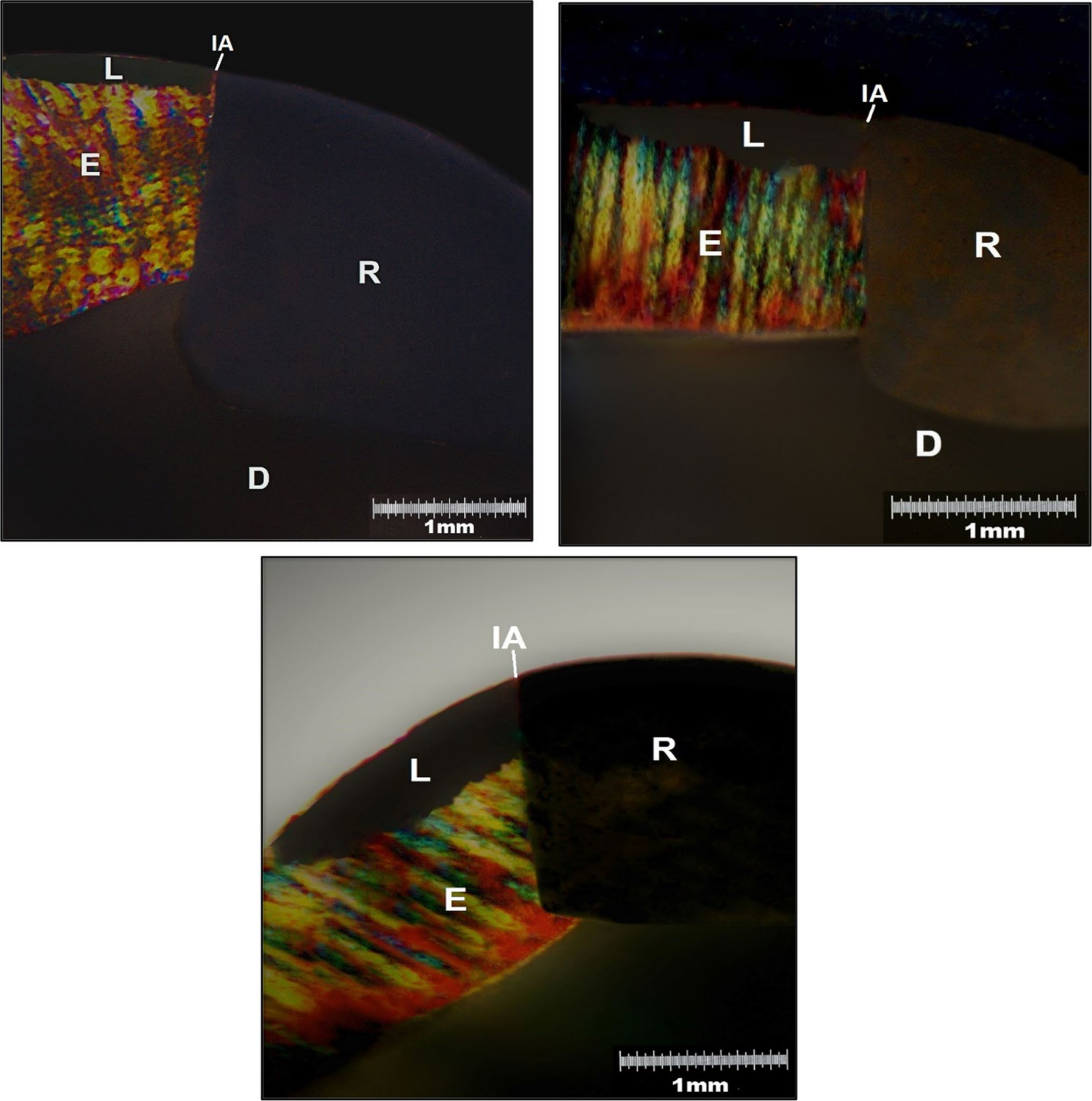
Microscopic images obtained by PLM. A: Occlusal lesions with positive birefringence (embedding in water) adjacent to Self-adhesive hybrid resin composite. Lesions were distant from the restoration, resulting in an inhibition area. B: Occlusal Lesion adjacent to Alkasite restorative material, with positive birefringence with inhibition area being visible. C: Occlusal lesions with positive birefringence adjacent to Bioactive flowable resin composite liner, with inhibition area that is very minimal. D: Dentin, E: Enamel, L: Lesion, R: Restoration, IA: Inhibition area.

Polarized light microscopy (10 × eyepiece lens and 2.5 × objective lens) was used to analyze photomicrographs of the teeth sections. Outer lesions and inhibition areas were seen next to restorations. No wall lesions were observed, Fig. 1.

Kruskal–Wallis H-test revealed a statistically significant difference in lesion parameters between the three materials. So, Pairwise comparisons were performed and revealed a statistically significantly higher extension and depth next to Bioactive flowable composite liner > Alkasite restorative material > Self-adhesive hybrid composite, and a statistically significantly smaller inhibition area in Bioactive flowable composite liner > Alkasite restorative material > Self-adhesive hybrid composite (Table 3).

**Table 3:** Comparisons of lesion parameters between the three materials (mm).

Parameter	Material						Pairwise comparisons of lesion parameters between materials
	Group A		Group B		Group C		
	Mdn	IQR	Mdn	IQR	Mdn	IQR	
Extension	1.36	1.31–1.38	1.75	1.69–1.78	1.54	1.47–1.57	A: < 0.001 B: 0.001 C: 0.001
Depth	0.27	0.23–0.29	0.56	0.54–0.57	0.40	0.39–0.43	A: < 0.001 B: < 0.001 C: < 0.001
Inhibition area	0.04	0.03–0.04	0.01	0.01–0.02	0.03	0.02–0.03	**A: < 0.001** **B: < 0.001** **C: < 0.001**

Notes: Group A: Self-adhesive hybrid resin composite. Group B: bioactive flowable composite liner. Group C: Alkasite restorative material Sig. = significance (p-value). IQR = interquartile range presented as Q1 and Q3. The test of significance is the Kruskal–Wallis H-test.

A: represent significant difference in Group A vs Group B, B: represent significant difference in Group A vs Group C, C:represent G significant difference in roup B vs Group c.

Significant values are in bold.

Spearman’s correlation test revealed a statistically significant strong negative correlation between fluoride release at each of the three time points vs. lesion extension and depth, and a statistically significant strong positive correlation between fluoride release at each of the three time points vs. lesion inhibition area. It also shows a statistically significant strong positive correlation between fluoride release at each of the three time points vs. fluoride release at the other two time points (Table 4).

**Table 4:** Correlation of fluoride release and lesion parameters.

Lesion parameter	Fluoride release					
	Day 1		Day 7		Day 14	
	r_s_	Sig	r_s_	Sig	r_s_	Sig
Extension	-0.882	**< 0.001**	-0.856	**< 0.001**	-0.826	**< 0.001**
Depth	-0.903	**< 0.001**	-0.853	**< 0.001**	-0.797	**< 0.001**
Inhibition area	0.857	**< 0.001**	0.850	**< 0.001**	0.796	**< 0.001**

Notes: r_s_ = Spearman’s correlation coefficient. Sig. = significance (p-value).

Significant values are in bold.

## Discussion

Ion-releasing dental materials combat bacteria and aid tooth remineralization, unlike bioinert resins composites^19^. Composite restoration failures occur due to biofilm buildup on resin surfaces and weak tooth-restorative interfaces, increasing secondary caries risk^36^. Therefore, dental materials’ bioactivity inhibits cariogenic bacteria and supports tooth bonding for biomineralization^37^.

The study utilized a novel Self-adhesive hybrid composite, an advancement from RMGIC, with high molecular weight polyacrylic acid, polymerizable groups, polyalkenoate acid copolymer, silanized non-reactive fillers, and FAS fillers. This material supports water and ion exchange in the oral cavity, releasing fluoride, aluminum, calcium ions, and potentially others^38^.

The Ion-releasing flowable composite contains Bio glass 45S5, releasing calcium, phosphate, and fluoride for tooth remineralization^39^. Low-viscosity flowable composites are ideal for small cavities, with easy syringe application that may reduce polymerization shrinkage through better stress relaxation^40^.

Alkasite restorative materials are analogous to GIC and RMGIC, where it has high fluoride liberation, as the company claims. Also, it acts as an esthetic filling material. Alkasite restorative material devours an alkaline filler, which may deliver acid neutralizing particles^41^.

To assess fluoride release, deionized water was chosen over saliva or pH-cycling due to its simplicity and ability to provide accurate fluoride release measurements without the potential interference of organic molecules or nutrients that might be present in saliva or pH-cycling solutions^42^. SPADNS colorimetric method was used in this investigation to measure fluoride release, as it is a simple and rapid technique with high accuracy^43^.

The significant initial release of fluoride from the Self-adhesive hybrid composite group on the first day might be attributed to an early discharge of fluoride from the glass components. This abrupt release of fluoride from the external surface into the surrounding liquid, coupled with the interaction between polyalkenoate acid and the glass components containing fluoride during the setting process, may elucidate the rapid burst in fluoride release^44^. The decline in fluoride release over the following days could result from the initial burst of fluoride being released from the glass particles as they dissolve within the polyalkenoate acid during the setting reaction. Furthermore, the reduction in fluoride release can be attributed to the diminished dissolution of glass particles within the material’s pores^45^.

The alkasite restorative material contains alkaline fillers that generate particles capable of neutralizing acids. When in a mixed state, the material consists of 23.5% alkaline glass by weight, potentially leading to an initial rise in fluoride release^46^. Based on the results of the present study, it was observed that the alkasite restorative material exhibited lower fluoride release compared to the Self-adhesive composite at all tested time intervals. This consistent fluoride release pattern in alkasite, without a burst effect, can be attributed to a higher powder-to-liquid ratio and a significant presence of alkaline glass in its final form.

This is consistent with a study that found that GICs emitted more fluoride than alkasite restorative material throughout all measurement intervals^47^.

H Abouelleil et al.^48^ found that the fluoride released amount was significantly higher for Self-adhesive hybrid composite on 14 days than alkasite restorative material which is in agreement with the current study. In contrast, another study has concluded that alkasite restorative material (self-cure) has the highest fluoride ion release and alkalizing potential in acidic pH as compared to GIC^49^.

Ion releasing flowable composite liner might have had lowest fluoride release because it lacks initial fluoride burst. Furthermore, unlike alkasite restorative material, it lacks alkaline fillers which might contribute to increased fluoride release. No previous studies were found for the Ion releasing flowable composite liner until the end of this study.

The study used closed batch culture for antibacterial assessment due to its ease, affordability, and minimal chemical requirements. Streptococcus Mutans, which is commonly used in caries models, was employed^50^. Previous studies have demonstrated that the use of a Streptococcus Mutans-based caries model for evaluating the demineralization-inhibiting effects of biomaterials was beneficial^51,52^. Selecting the best species and their relative abundances in multi-species models is a difficult task. Additionally, the use of a single-species model reduces the possibility of misleading aspects. The bacterial approach to in vitro artificial caries production has been employed in the current investigation because it allows the demineralization of dental tissue and enables the examination of various restorative material anti-cariogenic qualities or microleakage surrounding restorations. The bacterial approach is thought to replicate in vivo circumstances closely^53^. Furthermore, biological tests appear to be the most relevant tests of the in vitro experiments because clinical caries are most likely dependent on biofilm formation^54^.

The statistical analysis model employed in this study accounted for four distinct crown sites: occlusal, cervical, buccal, and lingual regions of the restorations. This approach was adopted due to the influence of various anatomical landmarks on the crown, which can impact the quality of the margin^24^. Polarized light microscope is employed to evaluate the inhibition area, lesion depth, and extension of enamel because it allows for better visualization of the histological features of enamel due to its inherent birefringence property. This characteristic, which is not effectively observed using a transmitted light microscope^55^.

Gram stain confirmed Streptococcus Mutans as the sole microorganism affecting the tested teeth. This microbiological technique distinguishes Gram-negative from Gram-positive bacteria based on dye retention due to cell wall properties. Streptococcus Mutans, a Gram-positive species, retained violet stain^24^.

The Self-adhesive hybrid composite showed the smallest outer lesion depth, and extension could be because the fluoride released affected outer (primary) surface lesions that occurred on enamel surfaces next to the restorations. When the fluoride is released from the adjacent restoration, the amount of minerals lost from the outer lesion is decreased. Lesion depth and mineral loss have a linear relationship with the total amount of fluoride produced over time^56^. Fluoride is known to decrease the production of bacterial acids and glucans by Streptococcus mutans, which is widely acknowledged as the primary causative factor behind the development of carious lesions^57^.

Alkasite restorative material presented higher resistance to secondary caries than Ion releasing flowable composite liner, as it released more fluoride and might have released more fluoride in the presence of bacterial acids and decreased pH. Alkasite restorative material might have released more fluoride in the existence of low pH due to dissolution of its surface-unaffected layer, which exposed more matrix for fluoride release^58^.

Ion releasing flowable composite liner had the least or no inhibition areas with largest lesion depth and extension, maybe due to the low amount of fluoride released at all time intervals. These results are in acceptance with a study has shown that inhibitory effects of fluoride on the growth of Streptococcus Mutans biofilms either in the early or mature stage were related to the concentration of the fluoride^59^. Another study have shown different results^60^. It demonstrated that demineralization inhibitory effect of alkasite restorative material is more than that of Self-adhesive hybrid composite, both in depth and in the degree of demineralization. These different results may be due to different techniques used in that investigation and different periods of time in which the study was conducted.

Chau et al.^61^ clearly demonstrate a negative correlation between the rate of fluoride release and various factors associated with the acid-producing capabilities, dry weight, bacterial bio-volume, and the amount of extracellular polysaccharides within Streptococcus mutans biofilms. These results strongly suggest that the release of fluoride from ion-releasing materials could be a pivotal factor in inhibiting the metabolic activity of cariogenic bacteria within biofilms. This suggests that the development of restorative materials capable of releasing higher concentrations of fluoride ions may lead to even more potent anti-cariogenic biofilm activity. Furthermore, such materials could potentially provide more efficient inhibition of subsequent secondary caries formation.

In a study Comparing fluoride-releasing sealants to non-fluoride-containing sealants, a notable decrease in the occurrence of wall lesions was observed. The mean outer lesion depths in enamel adjacent to fluoride-releasing sealants were considerably lower compared to non-fluoride-containing sealants^62^. On the other hand, Yap et al.^63^ reported that there was no antibacterial activity despite the presence of fluoride in the agar surrounding the test materials. However, the antibacterial effect can vary based on factors such as the size of the material’s diffusion, the shape of the filler forms, and the fluoride concentration in the material. Additionally, the release of ions is influenced by the pH of the medium.

## Limitations

Restorative materials were evaluated in laboratory conditions (in vitro study). The dynamic nature of conditions found in the oral cavity, such as salivary flow characteristics, presence of plaque, oral hygiene, and dietary habits utilized by the patient, which can lead to results that may be different from what has been proven in the current study. Thus, further studies employing clinical trials are important.

This study was conducted to evaluate the fluoride release of bioactive materials after 24 h and after short periods of time. So, there is a lack of information about fluoride release after longer periods of time. In addition, the caries inhibition behavior of the restorations was evaluated within enamel margins in class V. Subsequently, further studies are still needed to evaluate the inhibition of secondary caries with dentin margins.

## Conclusion

Within the limitations of this in vitro study, it could be concluded that:

1. The three ion-releasing restorative materials used in the current study exhibited fluoride release and prevention of secondary caries formation in direct contact with the restoration.
2. The Self-adhesive hybrid composite was the material with the highest fluoride-releasing potential at each time interval. Also, it was the material with the highest ability to inhibit secondary caries (in direct contact with the restoration), induced by cariogenic bacteria.
3. The Ion-releasing flowable composite liner showed the least fluoride release with no or minimal secondary caries inhibition.
4. All tested materials showed a decrease in fluoride release over time.
5. Increasing amount of fluoride release had led to increase the size of inhibition areas, while decrease the outer lesion depth and extension.

## Recommendations

- Further investigations are needed regarding fluoride recharge of the three bioactive restorative materials.
- Further studies are recommended to evaluate fluoride release within longer evaluation periods.
- Secondary caries inhibition ability of the tested materials could be investigated on restored teeth with a dentin margin to evaluate lesions and inhibition areas formation.
- Future studies are recommended with inclusion of a fluoride-free restorative material in the caries inhibition evaluation as a control to allow for direct comparison between fluoride-releasing materials and non-fluoride materials in terms of their ability to inhibit caries production.

## Data Availability

The datasets used and/or analyzed during the current study are available from the corresponding author on reasonable request.
